# The impact of sidewall copper grain condition on thermo-mechanical behaviors of TSVs during the annealing process

**DOI:** 10.1038/s41378-024-00830-1

**Published:** 2024-12-16

**Authors:** Yang Xi, Yunpeng Zhang, Zhiqaing Tian, Tianjian Liu, Can Sheng, Bo Zhao, Zhaofu Zhang, Shizhao Wang, Sheng liu

**Affiliations:** 1 The Institute of Technological Sciences, Wuhan University, Wuhan, 430072 China; 2 School of Mechanical Science and Engineering, Huazhong University of Science and Technology, Wuhan, 430074 China; 3 School of Power and Mechanical Engineering, Wuhan University, Wuhan, 430072 China

**Keywords:** Electrical and electronic engineering, Physics, Nanoscience and technology

## Abstract

With the drastic reduction of the TSV diameter leading to a critical dimension comparable to the Cu-filled grain size, the grain condition strongly influences the thermo-mechanical behavior of the TSV. In this work, the TSV-Cu cross-section with different grain sizes is characterized by EBSD, confirming that the sidewall grain size (0.638–1.580 μm) is smaller compared to other regions (1.022–2.134 μm). A finite element model (FEM) considering copper grains is constructed by using Voronoi diagrams to investigate the effect of sidewall grain size as well as area on the thermo-mechanical behavior during annealing. The material parameters in the FEM are optimized through nanoindentation inversion and considering the mechanical property anisotropy of copper grains. The yield strength *σ*_*y*_ and hardening exponent *n* of TSV-Cu are 74.6 MPa and 0.514. The simulation results indicate that the protrusion of TSV-Cu after annealing tends to increase initially and then decrease with smaller sidewall grain size and area. The maximum increase in protrusion caused by the two variables can reach 6.74% and 14.6%, respectively, relative to the average grain condition. Additionally, the simulation results were validated by quantifying grain boundaries in TSV-Cu samples with varying grain sizes.

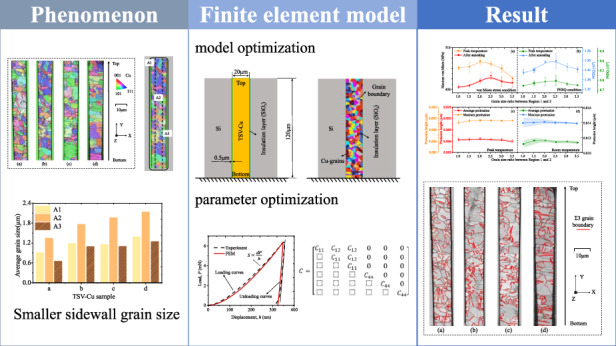

## Introduction

Though silicon via (TSV) technology has been widely used as one of the key technologies for realizing vertical electrical interconnections in 3D packaging. Copper (Cu) is widely adopted in TSV filling because of its excellent electrical and thermal conductivity^1,2^. However, the manufacturing processes involve high-temperature annealing treatments to eliminate the residual stresses. Due to the thermal mismatch between the major materials in TSVs (Cu is 16.7 ppm/°C, SiO_2_ is 0.7 ppm/°C, and Si is 2.6 ppm/°C), the copper is susceptible to protruding during the annealing process^3,4^, which potentially lead to irreversible failure^5^. Therefore, the mechanical behavior of TSV-Cu in a thermal environment becomes one of the main focuses of TSV reliability^5,6^.

Many factors affect the mechanical properties of TSV-Cu, such as electroplating parameters, annealing conditions, and process temperature^5,7^. The effect of annealing temperature^8,9^, TSV pitch^10^, and annealing temperature slopes^4^ has been investigated systematically. However, As the TSV size decreases dramatically, the grain size of TSV-Cu is comparable to the TSV size^11^, and the anisotropy between grains is more obvious along with the increase of the width-to-depth ratio^12^. Numerous related researches neglected to consider the construction of the copper grain.

To enhance simulation reliability and investigate the relationship between the microstructure and thermodynamic behavior, the phase field-crystal (PFC) method^13^, the phase-field method combined^6^, and the random grain model constructed by the Voronoi diagram^9^ were utilized. However, some of the material parameters still need to be optimized. Some previous studies used plasticity to characterize the copper mechanical behavior^5,9^ while this parameter was usually obtained from macroscopic copper material measurements. The micro-tensile method^14^ and the micro-compression method^15^ were used to determine the mechanical properties. However, the accuracy of the test results is difficult to guarantee due to the difficulty of sample fabrication. Li et al. introduced the nanoindentation inversion method to obtain the power-law constitutive model of TSC-Cu^16^. This method provides feasibility to measure TSV-Cu mechanical parameters.

Additionally, the influence of TSV-Cu grain condition on the protrusion has been widely studied. Researchers have investigated the effect of grain size from perspectives such as yield strength^4,15^, creep spreading rate^17^, and mechanical behavior^9,18^. Wu et al.^19^ and Zhang et al.^9^ investigated the influence of uneven distribution of average grain size in the vertical direction on thermo-mechanical behavior. An et al.^17^ pointed out statistically that the TSV-Cu grains in the sidewall region had smaller grain sizes than those in the middle. However, the copper material parameters in FEM models were still homogeneous. Therefore, the study of the effect of sidewall grains still lacks modeling work incorporating grain anisotropy and accurate elastoplastic mechanical parameters of the microscopic TSV-Cu structure.

In this work, TSV-Cu crystal distribution models with randomly generated grains are constructed by the Voronoi method. The power-law constitutive parameters of TSV-Cu are determined by nanoindentation experiments combined with the FEM inversion method. Based on the optimization model, the influences of the grain condition, especially the size and area of the sidewall grain, are investigated. The analysis focuses mainly on the thermo-mechanical reliability during the annealing process. The FEM simulation results are analyzed and verified by combining the microscopic characterization with different grain sizes. The present study aims to develop a more comprehensive understanding of the influences of axial differences in TSV-Cu grains on the mechanical behavior during the annealing process, which can provide new inspirations for improving structural reliability.

## Methods and materials

### Inversion algorithm and constitutive modeling

**Fig. 1:**
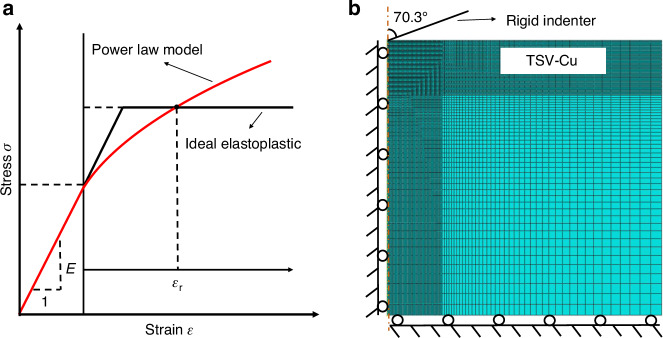
Power law constitutive and FEM model diagrams. **a** Elastoplastic constitutive model of TSV-Cu, **b** geometric model for FEM inversion

The power law constitutive model is widely used to describe the plastic behavior of metals and their alloys. Similarly, the plasticity characteristic of TSV-Cu can be effectively captured by utilizing this model. Figure 1a presents a complete description curve while its equation is shown in Eq. (1).

1 $$\left\{\begin{array}{l}\sigma =E\varepsilon \\ \qquad\sigma ={\sigma }_{y}{\left(1+\frac{E}{{\sigma }_{y}}{\varepsilon }_{p}\right)}^{n}\qquad\sigma\, < \,{\sigma }_{y}\end{array}\right.$$

where, *E*, *σ*_*y*_, and *n* represent Young’s modulus, yield strength, and hardening exponent, respectively, which are material-related. The determination of *σ*_*y*_ and *n* can adequately characterize the power law constitutive model.

Due to the inherent challenges in directly measuring the stress-strain curve of TSV-Cu, an indirect calculation method is employed, which combines the *P*–*h* curves from the nanoindentation tests with FEM inversion. As shown in Fig. 1b, a 2D symmetric model was used to simulate the nanoindentation tests, at which the indenter angle is converted to 70.3° (Ref. ^20^). The model was set to be axisymmetric and the bottom degree of freedom was fixed to ensure consistency with the boundary conditions. Mesh near the interface was subdivided in high density to ensure the calculation accuracy. Plasticity parameters were then defined using the power law constitutive model.

To determine the values of *σ*_*y*_ and *n* in Eq. (1), Dao et al.^21^ proposed the concept of characteristic stress *σ*_*r*_ and characteristic strain *ε*_*r*_. The simulation results will be consistent with the experimental data if using the identical Young’s modulus and share the same coordinate of (*ε*_*r*_,*σ*_*r*_), although they share different settings on stress-strain curves. Hence, the plasticity parameters can be determined by calculating the *σ*_*r*_, *ε*_*r*_, and *n*.

When Young’s modulus and *σ*_*r*_ are identical, the *P*–*h* curves in the loading stage will coincide and remain unaffected by *n*^22^. Therefore, the value of *σ*_*r*_ will be determined first. The initial value of *σ*_*r*_ can be estimated by solving Eq. (2) (Ref. ^23^):

2 $$\frac{{E}_{r}}{H}=0.231\left(\frac{{E}_{r}}{{\sigma }_{r}}\right)+0.491$$

where *H* is the hardness of the measured material, and *E*_*r*_ is the reduced modulus, both of which can be obtained by nanoindentation tests. The accuracy of *σ*_*r*_ necessitates improvement through the FEM model due to the unsatisfactory accuracy of the empirical formula. The *P*–*h* curves of the simulation result are obtained using the ideal elastoplastic (*n* = 0) FEM model and compared with the experimental *P*–*h* curves. Then, the value of *σ*_*r*_ should be iterated by the bisection method until the relative error between the simulation and the experimental loads at the maximum displacement is less than 0.5%.

The hardening exponent *n* can be calculated using the dimensionless function^21^, as shown in Eq. (3)

3 $$\frac{{h}_{r}}{{h}_{m}}=A{\left({\mathrm{ln}}\left(\frac{{\sigma }_{r}}{{E}_{r}}\right)\right)}^{3}+B{\left({\mathrm{ln}}\left(\frac{{\sigma }_{r}}{{E}_{r}}\right)\right)}^{2}+C{\mathrm{ln}}\left(\frac{{\sigma }_{r}}{{E}_{r}}\right)+D$$

where, *h*_*r*_ and *h*_*m*_ represent the residual depth and maximum depth in the nanoindentation experiments, respectively. The values represented by *A*–*D* are shown in Eqs. 4–7:

4 $$A=0.010100{n}^{2}+0.0017639n-0.0040837$$

5 $$B=0.014386{n}^{2}+0.018153n-0.088198$$

6 $$C=0.59505{n}^{2}+0.03407n-0.65417$$

7 $$D=0.58180{n}^{2}-0.088460n-0.67290$$

The value of the characteristic strain *ε*_*r*_ can be initialed by the empirical equation Eq. (8) (Ref. ^24^). Similarly, the value of *ε*_*r*_ can be determined by the same iterative method with FEM as *σ*_*r*_.

8 $${\varepsilon }_{r}={exp} \left(-3.19+166.7/\left({\sigma }_{r}/{\varepsilon }_{r}+177.3\right)\right)$$

Since the hardening exponent *n* can be uniquely determined from the unloading stiffness^23^, the value of *n* needs to be iterated constantly until the unloading slope of the simulation is consistent with the slope from the experiment.

Using the aforementioned method, the yield strength *σ*_*y*_ can be determined by substituting the values of *σ*_*r*_, *ε*_*r*_, and *n* into Eq. (1) to obtain Eq. (9). Finally, the power law constitutive equation of TSV-Cu can be determined.

9 $${\sigma }_{r}={\sigma }_{y}{\left(1+\frac{E}{{\sigma }_{y}}{\varepsilon }_{r}\right)}^{n}$$

### Modeling methodology

**Fig. 2:**
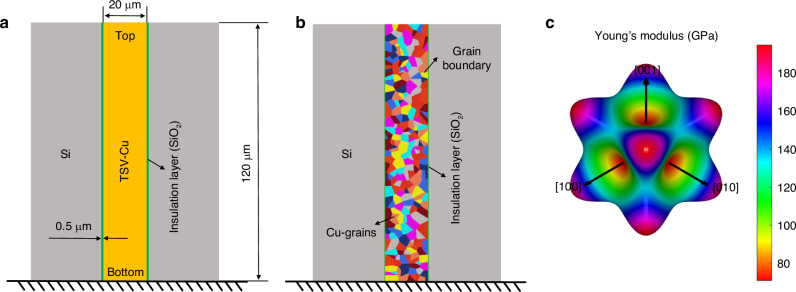
TSV simulation diagram and material properties diagram. **a** Cross-section configuration of a typical Cu-filled TSV structure, **b** FEM model considering random copper grains, **c** Cu FCC crystal Young’s modulus diagram in [111]

A typical two-dimensional model of Cu-filled TSV is considered in this work, as illustrated in Fig. 2a. The barrier layer is neglected due to its low relative thinness. As the diameter decreases, the dimensions of the copper grains gradually approach those of the TSV. Therefore, the consideration of the distribution of TSV-Cu grains should be considered. The elastic stiffness of copper grains is different with different orientations. The crystal structure of polycrystalline copper is face-centered cubic (FCC), and its elastic stiffness tensor can be expressed as Eq. (10):

10 $$C=\left[\begin{array}{cccccc}{C}_{11} & {C}_{12} & {C}_{12} & 0 & 0 & 0\\ & {C}_{11} & {C}_{12} & 0 & 0 & 0\\ & & {C}_{11} & 0 & 0 & 0\\ & & & {C}_{44} & 0 & 0\\ & & & & {C}_{44} & 0\\ & & & & & {C}_{44}\end{array}\right]$$

Where, *C*_11_ = 172.03 GPa, *C*_12_ = 121.97 GPa, *C*_44_ = 76.79GPa^25^. The tensor *C* enables the calculation of the elastic stiffness tensor *C’* for grains with a specific orientation^26^, as shown in Eq. (11):

11 $$C{\rm{\mbox{'}}}={MC}{M}^{T}$$

Where *M* is the transformation matrix associated with grain orientation^26^. This calculation procedure has been explained in previous studies^9^. The orientation-dependent Young’s modulus can be obtained from elastic stiffness calculations, as illustrated in Fig. 2c. There exist differences in Young’s modulus among grains of varying orientations, for instance, Young’s modulus in the <111> direction is about 2.8 times that in the <100> direction. Variations in mechanical parameters caused by the copper grains will have a non-negligible effect on the study of the mechanical properties. Therefore, the variation should be taken into the FEM model account.

FEM models considering copper grains were established, as shown in Fig. 2b, where different colors represent distinct grain orientations. The TSV diameter is 20 μm, while the depth of the blind hole is 120 μm. The thickness of the SiO_2_ insulator layer is 0.5 μm. The random distribution of grains was achieved by randomly distributing Voronoi points, and the grain size inside the TSV was simulated by controlling the number of Voronoi points. For grain orientation, 10 sets of copper grain with random orientations were selected^6,9^, and the grain regions were stochastically defined. The mechanical parameters of copper grains are detailed in Table 1. To simulate the high-temperature annealing process, the temperature field was configured to anneal at 400 °C in a trapezoidal profile. The material parameters utilized in simulations are listed in Table 2.

**Table 1:** Young’s modulus and Poisson’s ratio of grains with different orientations

Orientation	Young’s modulus (GPa)			Poisson’s ratio		
	*E*_1_	*E*_2_	*E*_3_	*v*_12_	*v*_23_	*v*_31_
1	191	133	150	0.362	0.442	0.156
2	72	93	93	0.416	0.250	0.536
3	108	105	113	0.409	0.361	0.354
4	92	112	128	0.444	0.201	0.476
5	94	71	109	0.446	0.418	0.227
6	136	79	147	0.445	0.389	-0.061
7	136	71	136	0.445	0.418	-0.091
8	143	127	173	0.484	0.275	0.229
9	156	112	164	0.445	0.352	0.105
10	135	144	181	0.441	0.198	-0.095

**Table 2:** Material parameters in the FEM model

Material	CTE (ppm/°C)	E (MPa)	*ν*	Plasticity
Cu	293 K:16.5 400 K:17.6 500 K:18.8	Details in Table 1		From experimental measurements
Si	300 K:2.62 400 K:3.26 623 K:4.40	130	0.28	–
SiO_2_	0.52	71	0.17	–

### Sample fabrication of TSV-Cu

**Fig. 3:**
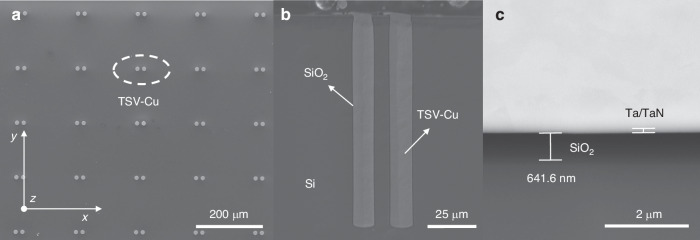
Schematic diagrams of TSV samples. **a** TSV arrays, **b** cross-section of TSV-Cu, **c** capped layers’ condition over the silicon substrate

Figure 3 shows the schematic diagrams of TSV samples, which commonly include the following steps: blind vias were firstly etched by deep reactive ion etching (DRIE) on a 12-in. Si wafer, and then a SiO_2_ insulator layer of 500–700 μm thickness was obtained by the thermal oxidation process. Ta/TaN-based barrier (Ta 300 nm, TaN 60 nm) and Cu seed layer (2000 nm) were deposited on the sidewall by physical vapor deposition (PVD). Subsequently, the blind vias were filled by Cu electroplating. Finally, chemical mechanical polishing (CMP) was used to flatten the surface and remove the excess copper. As shown in Fig. 3b, the depth and diameter of TSVs are 110 and 12 μm. The thickness of the SiO_2_ insulator layer is 641.6 nm, as shown in Fig. 3c. The thickness of the Ta/TaN barrier layer is almost negligible in comparison to SiO_2_, with a minimum thickness of approximately 5 nm.

### Characterization of mechanical properties and microstructure

The mechanical properties of TSV-Cu were measured by Nano Indentor (Nanotest Vantage). The constant loading and unloading rate was 20 μN/min, and the maximum displacement was 350 nm. Young’s modulus (*E*) and hardness (*H*) of the specimens were calculated based on the Oliver–Pharr method using the load–displacement curves, and four sets were measured repeatedly, and the results were averaged.

## Result and discussion

### Nanoindentation and constitutive inversion results

**Fig. 4:**
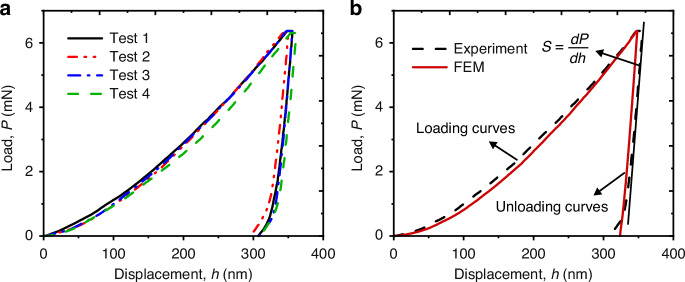
Nanoindentation test and FEM inversion results. **a** Nanoindentation curves of four repeated tests from TSV-Cu samples, **b** comparison of load–displacement curves between experiment and simulation

The results from the nanoindentation tests are shown in Fig. 4a, illustrating that the *P*–*h* curves of the four samples exhibit consistent trends with high repeatability. The measured values of Young’s modulus and hardness are 140.41 and 1.8047 GPa, respectively. The process of constitutive models for inversion was achieved based on the mechanical parameters obtained by nanoindentation experiments, and the *P*–*h* curves of experiment and simulation are shown in Fig. 4b. The initial stages of the unloading curves are nearly parallel, suggesting that the predicted results of the contact stiffness *S* are consistent with reality and that the value of *n* is accurate. The yield strength σ_y_ and hardening exponent *n* obtained by inversion of the simulation curves is 74.6 MPa and 0.514, respectively, which fall within the range of previous findings^16^ and are considered acceptable.

### Microstructural observation of TSV-Cu

**Fig. 5:**
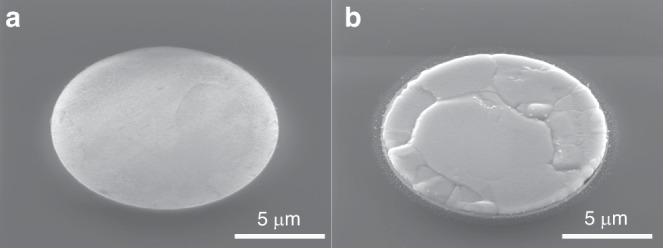
Protrusion morphologies of TSV-Cu. **a** Before annealing, **b** after annealing

The SEM images before and after annealing are shown in Fig. 5a, b. The top of the TSV samples was flat and smooth initially, but after annealing, the TSV-Cu exhibited a mushroom-like protrusion. Meanwhile, the protrusions likely contributed to delamination at the interface with SiO_2_, potentially leading to TSV failure.

**Fig. 6:**
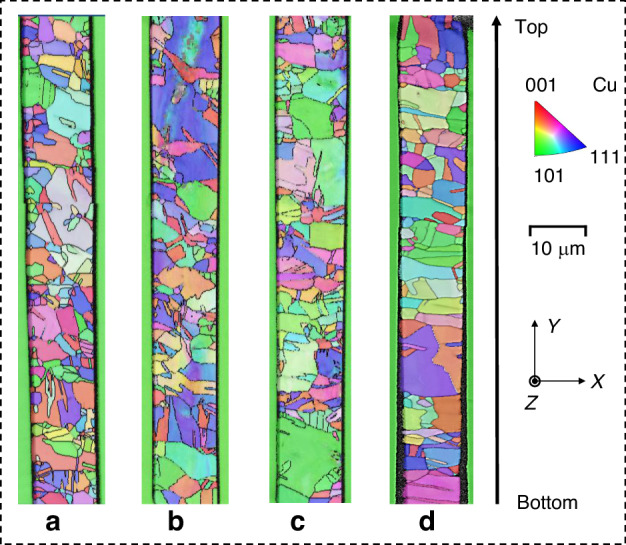
EBSD maps of TSV-Cu with various grain conditions. **a**–**d** Illustrate the TSV samples with sequentially increasing average grain size

Figure 6a–d presents the grain conditions characterized by EBSD. The average grain size of samples a–d increases sequentially, as shown in Table 3. The results illustrate that the distribution of copper grains inside the TSV is disordered. Furthermore, it is noticeable that the grain size appears smaller in the region near the TSV sidewall, primarily due to the presence of the sputtering Cu seed layer^17^.

**Table 3:** Average grain size of sample a–d

Specimens	Sample a	Sample b	Sample c	Sample d
Average grain size (μm)	1.063	1.453	1.547	1.726

**Fig. 7:**
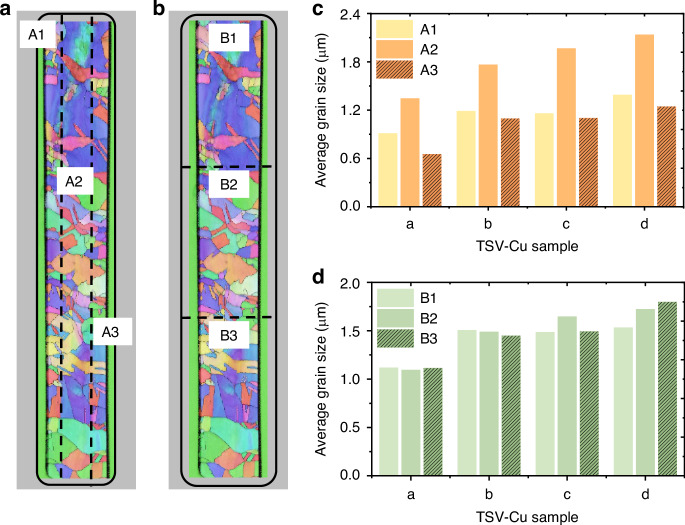
Average grain size statistics in different regions of TSV-Cu. **a** Statistical diagram along the axial direction, **b** statistical diagram along the longitudinal direction, **c** statistical result along the axial direction, **d** statistical result along the longitudinal direction

To concretize this phenomenon, the grain sizes of different regions are quantified, as shown in Fig. 7a, b. On the one hand, the results of the distribution along the axial direction are presented in Fig. 7c. The average grain sizes of samples a–d in the central region (A2, 6 μm × 110 μm) are 1.34 μm, 1.76 μm, 1.96 μm, and 2.13 μm, respectively. In contrast, the grain size in the sidewall region (A1, A3, 3 μm × 110 μm) is significantly smaller compared to the middle. The ratios of the average grain sizes in the sidewall and middle region range from 1/2 to 2/3. On the other hand, the results of the longitudinal grain distribution are shown in Fig. 7d. The grain sizes of the top, intermediate, and bottom regions (B1, B2, B3, 12 μm × 30 μm) shared unobvious trend and inconstant differences. Observations show that the TSV-Cu average grain differences are mainly along the axial direction.

### FEM analysis of the TSV-Cu annealing process

**Fig. 8:**
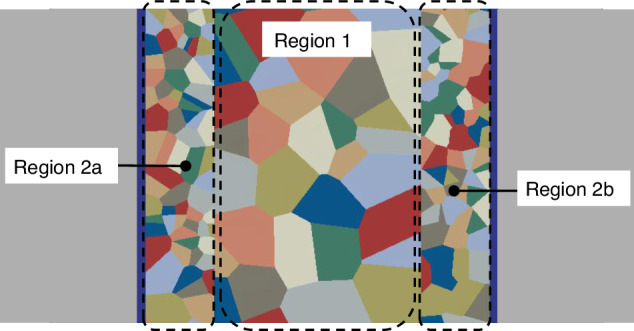
Part of the TSV FEM model considering polycrystalline anisotropic grain

According to the observation of the TSV-Cu morphology, the average size within the sidewall region is smaller than that within the central region. Based on the aforementioned FEM model in Section “Modeling methodology”, the TSV-Cu was delineated into the middle region (Region 1) and the sidewall region (Region 2), and the average size and area in the sidewalls were investigated, respectively, as illustrated in Fig. 8. The specific parameters of the simulation model are listed in Table 4. Considering the randomness of the model construction process, five sets of models were randomly generated for each type of variable to mitigate the influence of chance results, and the final results are presented as the average value.

**Table 4:** Parameters of FEM models

	Grain size of Region 1 (μm)	Grain size of Region 2 (μm)	Grain size ratio between Region 1 and 2	Grain area ratio between Region 1 and 2
Model S1	3.00	3.00	1.00	3.00
Model S2	3.00	2.00	1.50	3.00
Model S3	3.00	1.50	2.00	3.00
Model S4	3.00	1.20	2.50	3.00
Model S5	3.00	1.00	3.00	3.00
Model S6	3.00	0.86	3.50	3.00
Model R1	3.00	1.50	2.00	2.00
Model R2	3.00	1.50	2.00	3.00
Model R3	3.00	1.50	2.00	4.00
Model R4	3.00	1.50	2.00	5.00
Model R5	3.00	1.50	2.00	infinite

#### Mechanical behavior of TSV-Cu grain

**Fig. 9:**
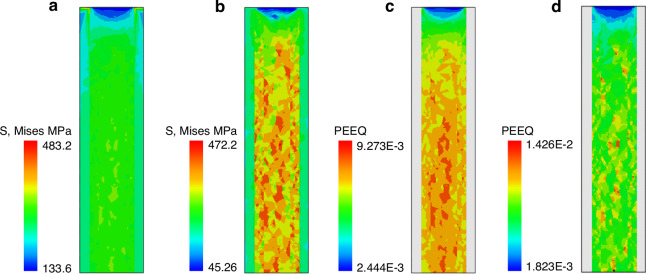
von Mises stress and PEEQ contour. **a** von Mises stress before annealing, **b** von Mises stress after annealing, **c** PEEQ before annealing, **d** PEEQ after annealing

During the annealing process, stress and strain are important mechanical parameters to consider. Figure 9 illustrates the von Mises stress and PEEQ (equivalent plastic strain) under the model S3 condition. When the annealing temperature reaches a maximum of 400 °C, the von Mises stress is obviously concentrated at the boundary where copper protrudes out, as shown in Fig. 9a. The stress concentration indicates that damage is more likely to occur here, which is observable in the SEM results. In the middle region at the top, the von Mises stress is less compared to other regions due to the stress relief achieved by copper expansion. At the middle and bottom areas, stresses are maintained at the same level due to the lack of stress release mechanisms. However, uneven stress distribution between different grains is observed in certain regions due to differences in mechanical parameters, as noted in previous experimental studies^27^. The PEEQ condition at the highest temperature shows significantly smaller plastic strain in the upper region compared to other areas, as illustrated in Fig. 9c. Despite similar stress magnitudes in the middle and bottom regions, there are noticeable differences in PEEQ among different copper grains due to variations in mechanical parameters.

The von Mises stress and PEEQ contour after annealing are shown in Fig. 9b, d, respectively. After cooling to 25 °C, the concentrated stress at the top is relieved by the cooling and retraction. Due to the plastic deformation in the copper region, the contraction is smaller than in other regions, resulting in the development of residual stress. Although the PEEQ condition remains consistent with the stresses, larger PEEQ values are generated in localized regions due to the smaller Young’s modulus of certain grains. Generally, these localized differences can lead to mutual extrusion, thus affecting the fatigue life^28^. Moreover, localized grain boundary sliding and grain boundary diffusion due to the deformation will develop cavities at TSV-Cu grain junctions, causing TSV failure^17^.

Figure 9 indicates that there is no significant difference in von Mises stress and PEEQ values between the sidewall and middle regions. Further analysis is required to understand the impact of the sidewall region on the annealing process.

#### Influence of sidewall grain size

**Fig. 10:**
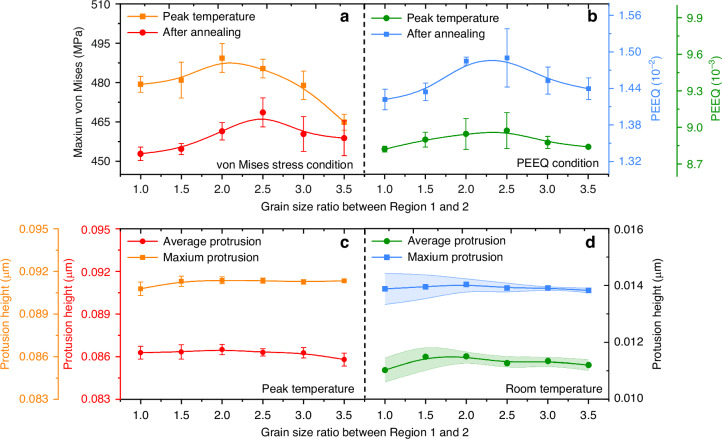
Effect of grain size on thermo-mechanical behavior of TSV-Cu at different annealing stages. **a** von Mises condition, **b** PEEQ condition, **c** copper protrusion at highest temperature, **d** copper protrusion at room temperature

To investigate the effect of sidewall grain size, models S1–S6 were constructed and analyzed comprehensively. Figure 10 depicts the trends in maximum von Mises stress, maximum PEEQ, and copper protrusion concerning sidewall grain size, both at peak annealing temperature and post-annealing. The simulation results reveal an initial rise followed by a gradual decline in maximum von Mises stress under both temperature conditions, correlating with an increase in the average grain size of Region 2. Compared with the model with uniform grain size, the models considering smaller grain size in the sidewall region have stress increases of 18.419 MPa and 23.713 MPa at the highest temperature and room temperature, respectively. This trend is further reflected in the upper and lower bounds of repetitive limits, as demonstrated in Fig. 10a. Figure 10b exhibits that the trend of PEEQ aligns consistently with the stress. The stress and PEEQ curves of the TSV-Cu model considering the grains consist of two distinct stages. On the one hand, the increase in the number of grains results in the presence of more grain boundaries, augmenting the number of neighboring grains with differences in mechanical parameters, which contributes to the difficulty of stress relief^6^. Consequently, the finer the grains, the finer grains are associated with elevated levels of stress and strain. On the other hand, according to the classic Hall–Petch (H–P) relationship and its derived formulas, the yield strength of the grain increases as the grain size diminishes, thereby enhancing the mechanical property strength under identical conditions^15^. Consequently, this improvement results in a more favorable stress and strain condition. Nevertheless, the trend of PEEQ is less pronounced during the highest temperature stage. The observed phenomenon can be attributed to the high strain state of the major grains at the maximum temperature, with PEEQ values predominantly dependent on temperature. As a result, minimal PEEQ variations are observed among the grains.

**Fig. 11:**
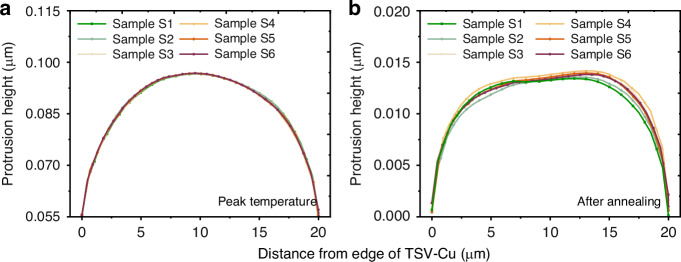
TSV-Cu protrusion distributions of models S1–S6 with the same intermediate grain condition. **a** At peak temperature, **b** after annealing

Figures 10c, d shows the maximum and average protrusion of TSV-Cu at the peak temperature and after annealing. The TSV-Cu protrusion distributions of models S1–S6 with the same intermediate grain condition are represented in Fig. 11. Based on Fig. 10c, the copper protrusions of TSV exhibit minimal correlation with the grain size of Region 2 and remain consistent at the highest temperature stage. Additionally, the protrusion of Region 1 is notably greater than that of Region 2, as shown in Fig. 11a. This discrepancy arises from the thermal expansion of copper. The copper expansion in sidewall areas is restricted by the comparatively slight protrusion of the silicon near the sidewall of TSV. Expansion curves of models S1–S6 reveal a remarkable degree of alignment, despite variations in grain sizes of Region 2. This result is due to the predominant influence of temperature on copper protrusion at peak temperatures. Therefore, the grains in the sidewall region are pulled by the intermediate region, leading to similar deformation.

The effect of sidewall grain size on the copper protrusion becomes increasingly obvious when the temperature drops back to room temperature, as shown in Fig. 10d. In comparison to Model S1, the copper protrusion initially rises to 6.735% (Model S3) followed by a reduction to −0.5% (model S6) as the sidewall grain size decreases, which aligns consistently with the trend of stress and strain. The protrusion curves depicted in Fig. 11b indicate that the expansions of both sides of TSV-Cu gradually surpass that of the middle region, resulting from the gradual contraction of copper during the cooled period, and the maximum protrusion occurs in the sidewall region. This observation is consistent with findings from prior simulation studies^18^. This phenomenon is mainly caused by the presence of more plastic deformation in the smaller grains. Additionally, it can be visualized in Fig. 11b that the values variation of the copper protrusion differs across different regions, and the larger variation appears in the sidewall regions. The average standard deviation of the protrusion difference in Region 1, Region 2a, and Region 2b are 3.99e−4, 2.48e−4, and 6.35e−4, respectively. It can be concluded that the protrusion after annealing is primarily determined by the sidewall grain, with potential influence extending to the middle region as well. Two distinct protrusion stages are also observed with sidewall grain size. In the initial stage, as copper grain size decreases, the copper filler becomes more prone to plastic deformation due to the more serious stress condition caused by the increase in grain boundaries, which eventually leads to an increase in protrusion. Such a trend has been confirmed by previous experimental studies^29,30^. In the second stage, the homogenization of parameters between sidewall grains occurs with the reduction in grain size. Since the protrusion is strongly dependent on the grains near the top^9,29^, the homogenization will result in a decrease in the copper protrusion, which is consistent with findings from previous studies^31^. This parameter homogeneity also effectively explains the phenomenon that smaller sidewall grain sizes correlate with less margin of error in the simulation results, as illustrated in Fig. 10d.

#### Influence of sidewall grain area

**Fig. 12:**
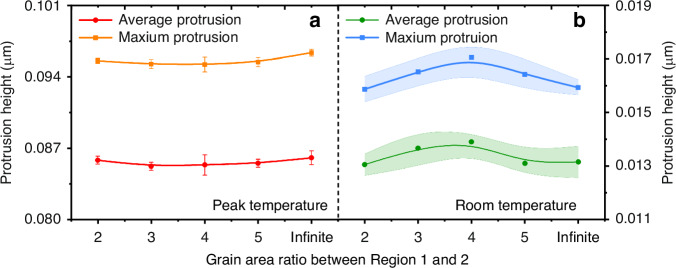
Effect of grain area on copper protrusion at different annealing stages. **a** At peak temperature, **b** at room temperature

To investigate the influence of the distribution range of smaller grains in the sidewall, models R1–R5 were constructed using the parameters shown in Table 4. Figure 12a demonstrates the trend of the protrusion with the range of sidewall grains at the highest temperature and after annealing. At the highest temperature stage, both the maximum and average protrusion values exhibit a slight decrease initially and then increase with the expansion of the sidewall grain range. However, considering the presence of errors, it can be assumed that the swelling value is independent of the sidewall grain range. As the temperature cools down to room temperature, both the maximum and average protrusion of copper initially increase, then decrease as the sidewall grain range expands, as shown in Fig. 12b. Notably, when the ratio of the intermediate grain range to the sidewall grain range is 4:1 (model R3), there is a significant 14.615% rise in maximum protrusion compared to the uniform grain model (Model R5), which may result in heightened damage severity. This phenomenon is a result of the increase in grain boundary length caused by the expansion of the sidewall grain area, thereby influencing the overall expansion process. The effect of grain area on protrusion is more pronounced than that of grain size.

### Influencing mechanisms of TSV-Cu grain

**Fig. 13:**
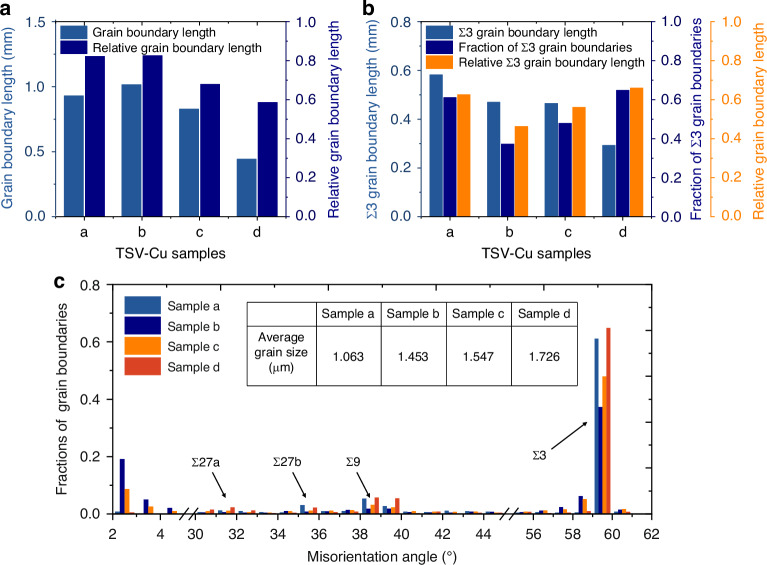
Statistic analysis of TSV-Cu grain boundary condition. **a** Grain boundary length, and relative grain boundary length **b** Σ3 grain boundary length, a fraction of Σ3 grain boundaries, and relative Σ3 grain boundary length, **c** specific fractions of grain boundaries

To figure out the influence of copper grain condition on TSV-Cu protrusion, EBSD results were analyzed and quantified. Grain boundaries are the main source of defects^8^ and are among the main factors contributing to grain boundary diffusion^32^ during annealing, ultimately leading to copper protrusion. Figure 13a shows the count of grain boundary length in the cross-section of TSV-Cu samples a–d. Due to the deviation of the measured areas among different samples, the relative grain boundary length (the ratio of the grain boundary length to the area of the measured region) was also counted. The grain boundary length and the relative grain boundary length increase as the average grain size increases from 1.063 μm to 1.453 μm. When the average grain size further increases to 1.726 μm, both the grain boundary length and the relative grain boundary length decrease. This phenomenon indicates that grain boundary length initially increases and then decreases as the average grain size diminishes, resulting in an initial increase followed by a decrease in the protrusion of TSV-Cu. The observation remains consistent with the prediction obtained from the simulation.

**Fig. 14:**
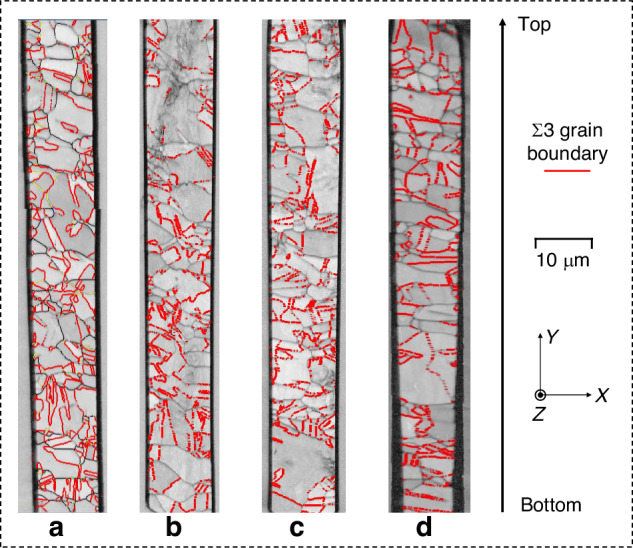
Distribution of Σ3 grain boundaries with various grain conditions. **a**–**d** illustrate the TSV samples with sequentially increasing average grain size

Among the grain boundaries, the coincidence site lattice (CSL) grain boundary represents the ideal twin boundary with specific orientation angles. This type of twin grain boundary has lower energy as well as lower energy mobility, making it more difficult to induce grain boundary cracking, corrosion, and deformation^33^. In the CSL grain boundaries, Σ3-twin grain boundaries can provide critical energy barriers for copper grains with lower intrinsic stacking fault energies^33^, thus preventing slip transfer between adjacent grains. Therefore, the fraction of Σ3 grain boundaries will affect the grain protrusion during annealing. Figure 14 demonstrates the distribution of Σ3 grain boundaries in the cross-section of samples a–d, where Σ3 grain boundaries account for a large proportion of the total grain boundaries. The specific fractions of different CSL grain boundaries are detailed in Fig. 13c. Notably, Σ3(60° <111>) grain boundaries hold the highest fraction of more than 40%, while the fractions of rest prominent grain boundaries, i.e., Σ9(38.94° <111>), Σ27a(31.58° <111>), Σ27b(35.42° <111>), are below 10%. The variation of Σ3 grain boundaries is particularly pronounced with different average grain sizes and thus may have a greater potential impact. Figure 13b statistically shows the Σ3 grain boundary length, fraction of Σ3 grain boundaries, and relative Σ3 grain boundary length (ratio of Σ3 grain boundary length to total grain boundary length). It can be seen that the Σ3 grain boundary length decreases with increasing grain size, which is attributed to the decrease of the total grain boundary length. The fraction of the Σ3 grain boundary, as well as the relative grain boundary length, both decrease and then increase with increasing grain size. This phenomenon indicates that both larger and smaller grains exhibit higher resistance to protrusion, aligning with the trend observed in the simulations. Table 5 shows the proportion of Σ3 grain boundaries in different regions of samples a–d. The fraction of the Σ3 grain boundary in the middle region is generally higher than that in the sidewall regions, although a few samples show a higher fraction in the sidewall regions. This result indicates that the intermediate grains are less prone to deformation during the swelling process, which is consistent with the protrusion curves obtained from the simulations.

**Table 5:** Proportion of Σ3 grain boundaries in different regions of samples a–d

Specimens	Average grain size (μm)	Fraction of Σ3 grain boundary		
		Region A1	Region A2	Region A3
Sample a	1.063	0.5202	0.7241	0.4760
Sample b	1.453	0.3546	0.4477	0.5373
Sample c	1.547	0.4331	0.6012	0.4712
Sample d	1.726	0.6748	0.6309	0.6125

**Fig. 15:**
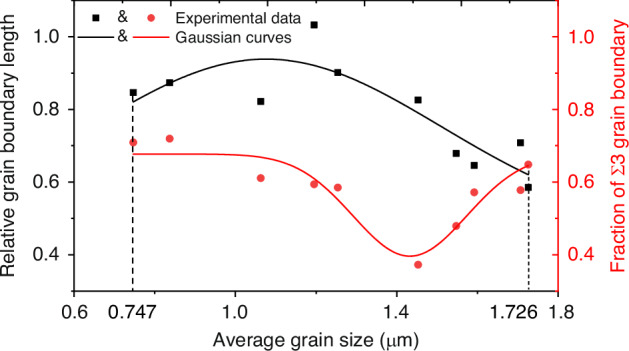
Statistical diagram of relative grain boundary length and fraction of Σ3 grain boundary with varying average grain size

To enhance the credibility of the aforementioned conclusions, the number of experimental samples was expanded to 10 groups, as illustrated in Fig. 15. The experimental results show that the relative grain size and fraction of Σ3 grain boundaries reach their maximum and minimum values when the grain size is in the range of 1.20–1.45 µm, respectively. The statistical trends display a parabolic pattern, aligning with the conclusions presented in Fig. 14. Based on the EBSD results, it is determined that when TSV average grain sizes are between 0.747 and 1.726 μm, the average sidewall grain sizes range from 0.638 to 1.580 μm, while the intermediate grain sizes range from 1.022 to 2.134 μm. Consequently, the intermediate grain size is positioned to the right of the parabolic curve illustrated in Fig. 15, indicating a gradual stabilization of the intermediate grain protrusion. In contrast, the sidewall grain size is distributed on both sides of the parabolic peak. This phenomenon indicates that sidewall grain is the main factor affecting the parabolic variation in protrusion.

Annealing experiments were carried out to verify the accuracy of grain size influence trend on protrusion. Following the principle of grain growth during annealing^8,9,34^, TSV samples with varying grain sizes were prepared by annealing wafers in batches for different durations, followed by CMP to obtain flat surfaces. The procedure is designed based on the dual annealing process employed in the production of TSV. Protrusion measurements were then taken using AFM after annealing the treated samples for the same duration. The experimental parameters and results are detailed in Table 6. There is one CMP process for every two annealing processes. The experimental results show that the TSV maximum protrusion after last annealing increases from the initial 15 nm to 202 nm, with the grain growth (total preparation annealing time from 0 h to20h). Subsequently, with the grain continuing to increase (total preparation annealing time from 20 h to 40 h), the maximum protrusion gradually decreases to 46 nm. This phenomenon verifies the results of simulation and analysis.

**Table 6:** Statistics of sample expansion with different annealing times

Number	First annealing	Second annealing	Last annealing	Final maximum protrusion (nm)
1	None	None	250 °C, 8.5 h	15 nm
2	250°C, 8 h	250°C, 8 h		50 nm
3	250°C, 16 h	250°C, 16 h		60 nm
4	250°C, 20 h	250°C, 20 h		202 nm
5	250°C, 24 h	250°C, 24 h		57 nm
6	250°C, 40 h	250°C, 40 h		46 nm

## Conclusions

In this study, the nanoindentation inversion method is employed to construct the power law constitutive model of TSV-Cu. The general phenomenon of smaller grain sizes in the sidewall region is characterized by observing the microstructure of TSV-Cu samples with different average grain sizes. The influence of the sidewall grains on the mechanical properties of TSV-Cu during annealing is investigated through FEM models, which are optimized using a stochastic grain model based on Voronoi diagrams. Furthermore, these FEM models consider the anisotropy of the mechanical properties of copper grains. The simulation results are interpreted based on the microstructure characteristic of TSV-Cu. The main results are summarized as follows:

1. The power law constitutive model of TSV-Cu is determined by the method that combines the FEM inversion with nanoindentation. The measured value of elastic modulus is 140.41 GPa, and the fitted values of yield strength *σ*_*y*_ and hardening exponent *n* are 74.6 MPa and 0.514, respectively.
2. The sidewall grains have a significant lateral effect on the mechanical behavior, especially the protrusion condition of TSV-Cu during annealing. The decrease in the average size and area leads to an increasing and then decreasing trend in the protrusion after annealing. In this work, the maximum deviation in protrusion relative to the average grain model, induced by sidewall grain size and range variables, can reach up to 6.735% and 14.615%, respectively. The copper protrusion at the highest temperature is almost impervious to the change of sidewall grains.
3. The grain boundary parameters, including the relative grain boundary length and the fraction of the Σ3 grain boundary, reach their maximum and minimum values when the grain size is in the range of 1.20–1.45 µm, respectively, with parabolic trends. This trend can reasonably explain the simulation results. Protrusion measurements verified the analytical results. Accompanied by grain growth, the TSV maximum protrusion shows a variation of 15 nm–202 nm–46 nm after the same annealing time.

This study can provide some guidance on enhancing the manufacturing process of high-reliability TSV. Through the optimization of manufacturing processes and parameters to achieve the minimal or maximal sidewall grain size and range, it is possible to mitigate the thermal-mechanical behaviors of TSV when subjected to thermal loading.
